# Hyperinsulinemia Induced Altered Insulin Signaling Pathway in Muscle of High Fat- and Carbohydrate-Fed Rats: Effect of Exercise

**DOI:** 10.1155/2021/5123241

**Published:** 2021-02-23

**Authors:** Anu Joseph, S. Parvathy, Koyikkal Karthikeya Varma

**Affiliations:** MIMS Research Foundation, Mankavu P.O., Calicut, Kerala 673007, India

## Abstract

Insulin resistance is a state of impaired responsiveness to insulin action. This condition not only results in deficient glucose uptake but increases the risk for cardiovascular diseases (CVD), stroke, and obesity. The present work investigates the molecular mechanisms of high carbohydrate and fat diet in inducing prediabetic hyperinsulinemia and effect of exercise on InsR signaling events, muscular AChE, and lactate dehydrogenase activity. Adult male Wistar rats were divided into the control (C) diet group, high-carbohydrate diet (HCD) group, high-fat diet (HFD) group, and HCD and HFD groups with exercise (HCD Ex and HFD Ex, respectively). Acetyl choline esterase activity, lactate dehydrogenase activity, total lactate levels, IRS1 phosphorylations, and Glut4 expression patterns were studied in the muscle tissue among these groups. High carbohydrate and fat feeding led to hyperinsulinemic status with reduced acetylcholine esterase (AChE) activity and impaired phosphorylation of IRS1 along with increased lactate concentrations in the muscle. Exercise significantly upregulated phosphoinositide 3 kinase (PI3K) docking site phosphorylation and downregulated the negative IRS1 phosphorylations thereby increasing the glucose transporter (GLUT) expressions and reducing the lactate accumulation. Also, the levels of second messengers like IP3 and cAMP were increased with exercise. Increased second messenger levels induce calcium release thereby activating the downstream pathway promoting the translocation of GLUT4 to the plasma membrane. Our results showed that the metabolic and signaling pathway dysregulations seen during diet-induced hyperinsulinemia, a metabolic condition seen during the early stages in the development of prediabetes, were improved with vigorous physical exercise. Thus, exercise can be considered as an excellent management approach over drug therapy for diabetes and its complications.

## 1. Introduction

Type 2 diabetes mellitus is a complex metabolic disease with an environmental and genetic component affecting over 5% of the population in Western societies. Recent data from the World Health Organization Multinational Study of Vascular Disease in Diabetes indicate that the main cause of mortality (52% of deaths) in type 2 diabetes patients is due to CVD [1, 2]. Several studies suggest 3 phases for type 2 diabetes mellitus development: hyperinsulinemia stage, prediabetes stage, and diabetes stage [3]. While most of the studies focus on the diabetes stage, the prevalence of hyperinsulinemia and associated multiple abnormalities is less studied. Approximately one-third of ingested glucose is used by the liver and the rest by peripheral tissues, primarily the skeletal muscle via an insulin-dependent mechanism. High glucose stimulates the pancreas to secrete more insulin thereby increasing the high glucose uptake in the skeletal muscle leading to the clearance of ingested glucose. In insulin resistance states, insulin-stimulated glucose disposal in the skeletal muscle is markedly impaired. In the transition from normal to impaired and diabetic glucose tolerance, hyperinsulinemia is the initiating agent which exists long before type 2 diabetes occurs, and an intervention or treatment during this early stage/hyperinsulinemic period may have a better opportunity to prevent or delay the occurrence or development of diabetes and its complications. The harmful effect of insulin on the vascular endothelium has been supported by several animal and human experimental studies. Insulin has several actions that may be related to the development of atherosclerosis [4, 5].

Skeletal muscle insulin resistance is considered to be the initiating defect that develops before *β*-cell failure and hyperglycemia becomes evident. In muscles, insulin resistance is associated with impaired insulin action on glucose transport and glucose metabolism. Insulin binding to cell surface insulin receptor (InsR) initiates a complex series of intracellular signaling events that lead to the numerous cellular effects of insulin including increased glucose uptake and glycogen synthesis [6–8]. At the molecular level, decrease in the glucose uptake is linked to a reduced tyrosine phosphorylation along with the involvement of serine phosphorylation of insulin receptor substrate 1 (IRS1). This shows equilibrium between “positive” tyrosine and. “negative” serine phosphorylation which is necessary for the IRS1 downstream functions [9, 10]. Insulin resistance has a serious effect on most of the properties of skeletal muscles including the changes in neuromuscular transmission. Of all the cholinergic synapses in the central and peripheral nervous systems, acetylcholinesterase (AChE) is an important component which rapidly hydrolyses acetylcholine released from the nerve terminals [11]. Patients with insulin resistance have increased activity of glycolysis leading to increased production of NADH and pyruvate. The conversion of pyruvate to lactate by lactate dehydrogenase (LDH) may be exaggerated during increased glycolysis driven by hyperinsulinemia [12]. Studies have shown that high lactate concentrations in tissue may inhibit the further glycolysis resulting in impaired glucose utilization [13].

Regular exercise represents an effective strategy to prevent and treat type 2 diabetes, decreasing glycemia. Although the mechanisms by which insulin signaling is enhanced after exercise remain unclear, the gene expression of key proteins involved in the insulin signaling pathway could be mediated by endurance training and may account for some of the improvements in insulin signaling after chronic exercise [14–18]. Endurance training is known to increase neuromuscular surface area, synaptic folds, and acetylcholine receptor numbers and may improve the release of Ach and activity of AChE [19].

The present study was done as sequelae to our clinical study which showed a high association between coronary artery disease and high insulin resistance and high fasting plasma insulin even in the absence of hyperglycemia or diabetes [20]. Because the skeletal muscle is the predominant site of insulin-mediated glucose uptake, the current study was done to explain the mechanism of hyperinsulinemia and differential effects of exercise on the insulin signaling pathway and also the metabolic dysregulations during hyperinsulinemia induced by high carbohydrate and fat diets in skeletal muscles.

## 2. Materials and Methods

All animal care and procedures were in accordance with the recommendations in the Guide for the Care and Use of Laboratory Animals by the Committee for the Purpose of Control and Supervision of Experiments on Animals (CPCSEA), Govt. of India, and was approved by the MIMS Research Foundation Ethics Committee.

### 2.1. Animals

The type of rats used, diet, and methods of estimating body weight, blood glucose, and total insulin levels were as described by Joseph et al. [21–23]. Adult male Wistar rats were purchased from Sree Chitra Tirunal Institute for Medical Sciences and Technology, Thiruvananthapuram, Kerala, and used for all experiments. 180–200 g body weight was maintained at their housing conditions with a controlled humidity (55%) and a 21 ± 1°C temperature, under 12-hour light and 12-hour dark periods and were maintained on standard food pellets and water *ad libitum*. Control, high-carbohydrate, and high-fat feed were purchased from the Animal Nutrition Department, Kerala Veterinary and Animal Sciences University, Mannuthy, Kerala, comprising 18.6% proteins, 44.2% carbohydrates, and 6.2% fat for the control group. High-fat diets contained as percentage of calories 18% proteins, 24% carbohydrates, and 58% fat, and high-carbohydrate diets, 21.8% proteins, 75.6% carbohydrate, and 2.5% fat.

After a week of acclimatization, the rats were randomly grouped into five groups with 10-12 animals in each group.

- Control group (control diet): C
- High-fat group (58% high-fat diet): HFD
- High-carbohydrate group (75.6% high carbohydrate): HCD
- HFD with exercise group (HFD+30 minutes exercise for two weeks): HFD Ex
- HCD with exercise group (HCD+30 minutes exercise for two weeks): HCD Ex

The control group was fed a standard diet, and the other four groups received a high fat or carbohydrate diet. After 4 weeks, for the exercise groups HFD Ex and HCD Ex, rats were randomly chosen from the HFD and HCD groups, respectively, and trained on a small animal treadmill running at 20 m/min for 30 min, 5 days/wk. Diet was maintained for 6 weeks. All rats were familiarized to the treadmill by walking 10 m/min for 10 min on the first day, then slowly increasing the duration to 20 min on the second day, and then 30 min on the third day. After becoming familiarized with treadmill running, the rats were trained at 20 m/min for 30 minutes [24]. Blood glucose, total circulating insulin, and body weight were monitored and recorded periodically. At the end of 6 weeks, fasting blood was collected from the vena caudalis for biochemical analysis. The rats were then weighed and sacrificed by intraperitoneal barbiturate overdose (200 mg/kg) after an overnight fast and 48 h after the last training session in the case of the trained rats. A detailed study timeline and an exercise protocol were included as Suppl. Figure I and Suppl. Table I. Under sterile conditions, tissues were dissected and stored at −80°C for further analysis. Glucose and cholesterol levels were determined by commercial colorimetric kits (Span Diagnostics), and total circulating serum insulin was estimated using an Insulin ELISA kit (Mercodia) according to the manufacturer's instructions. Skeletal muscles (vastus lateralis and Gastrocnemius muscles) were used for further analysis.

### 2.2. Acetylcholinesterase Assay in Muscles: Tissue Preparation

One gram of each muscle tissue was homogenized using a sodium phosphate buffer (0.1 M, pH 8) at a ratio of 1 part of tissue to 9 parts of buffer at 4°C. The homogenate was then centrifuged at 9000×g for 5 min at 4°C.Supernatant was collected, and protein was estimated by Bradford method and stored at -20°C. Approximately 300 *μ*g of protein was used for further kinetic assay. Enzyme activity was measured using Ellman's method [25]. The reaction mixture contained 100 mM phosphate buffer pH (7.5), 0.75 mM 5,5′-dithio-bis-2-nitrobenzoic acid (DTNB), and the enzyme (300 *μ*g of protein) and was preincubated for 2 min. After 2 min at 25°C, the reaction was initiated by adding acetylthiocholine iodide. Varying concentrations of acetylthiocholine iodide 0.5 to 5 mM were used. The method is based on the formation of a yellow anion, 4,4′-dithio-bis-nitrobenzoic acid, measured by absorbance at 412 nm. The enzyme activity was expressed as *μ*mol AChE hydrolyzed/h per mg of protein. The results of rate measurements as a function of acetyl choline concentration are illustrated by the graphs, which show data obtained at 30 min incubation at 37°C. Specific activity (SA) versus acetyl choline concentration relationships were analyzed in terms of the Michaelis–Menten graph. The maximal reaction rates (*V*_max_) and the Michaelis–Menten constants (*K*_*m*_) were calculated from the graph.

### 2.3. Kinetic Analysis of Lactate Dehydrogenase

Lactate dehydrogenase catalyzes the conversion of lactate to pyruvate, the forward reaction, and the conversion of pyruvate to lactate, the reverse reaction. LDH activity in muscles was assayed spectrophotometrically by the method of Pesce et al. [26]. NADH strongly absorbs light at 340 nm, whereas NAD^+^ does not. The rate of increase in absorbance at 340 nm is directly proportional to the lactate dehydrogenase activity in the sample. The muscle tissue was excised and was minced in Tris- HCl buffer (0.2 M, pH 7.3). The homogenate was then centrifuged, and the supernatant was taken. The protein estimation was done by the Bradford assay. Approximately 100 *μ*g of protein was used for further kinetic assay. Lactate dehydrogenase activity was assayed spectrophotometrically at 340 nm using 1.5 mM pyruvate and 0.5 mM NADH as the substrate and cofactor, in 0.2 M Tris-Cl buffer, pH 7.4. For kinetic determinations, a variety of substrate pyruvate concentrations (0.5 mM to 2.5 mM) were used. All kinetic experiments were designed for the conversion of the pyruvate to lactate. The rates of oxidation of NADH were carried out for different time intervals. The maximal reaction rates (*V*_max_) and the Michaelis–Menten constants (*K*_*m*_) were calculated from the graph.

### 2.4. Determination of Lactate Concentration

Muscle tissue was homogenized in lactate assay buffer and centrifuged at 13,000 g for 10 min to remove insoluble material. Lactate levels in the tissue were performed using a lactate assay kit (Cat. no. MAK064; Sigma-Aldrich; Merck) according to the manufacturer's protocol.

### 2.5. Estimation of Rat IP3, Diacylglycerol Acid, Cyclic AMP, and Cyclic GMP

Levels of the IP3, DAG, cAMP, and cGMP were determined by enzyme immunoassaying following the protocol as described by the supplier's manual (IP3 ELISA Kit, Cusabio Biotech; DAG ELISA Kit, Bioassay Technology Laboratory; cAMP, cGMP ELISA Kit, Invitrogen).

### 2.6. Western Blotting

The muscle tissues were lysed on ice in a RIPA lysis buffer supplemented with protease inhibitors and phosphatase inhibitor and 1 mM PMSF. After homogenization, the preparation was centrifuged at 20,000 g for 20 minutes at 4°C. The supernatant was then stored at -80°C for later analysis. The protein content in each sample was measured by the Bradford method. Equal amounts of lysate proteins (40 *μ*g) were separated in 10% SDS PAGE gels before being transferred to polyvinylidene fluoride membranes. The membranes were blocked with 5% skim milk and then immunoblotted with antibodies 1 : 1000 anti-InsR, 1 : 1000 anti-IRS1 (phospho-Y612), 1 : 1000 anti-GLUT4, 1 : 500 pIRS1 (Ser 636), and 1 : 500 pIRS1 (Ser 639) (Santa Cruz) overnight at 4°C. After washes with 0.1% Tween 20 in TBS, the membranes were incubated with a horseradish peroxidase-linked secondary antibody. The protein bands were visualized after developing with a BCIP/NBT reagent. Protein content was normalized by monoclonal anti-*β*-actin (1 : 2000, Sigma-Aldrich).

### 2.7. Statistical Analysis

Data are presented as mean ± SE. Statistical analysis was conducted using GraphPad Prism 5.0 software (GraphPad Software Inc., USA). Statistical evaluation was done by 2-way ANOVA. Differences between groups were considered statistically significant if *P* < 0.05.

## 3. Results

### 3.1. Standardization of Hyperinsulinemic Rat Model

Our studies showed that body weight, total cholesterol, and total circulating insulin of high-fat-and high-carbohydrate-fed rats increased compared to those of the control (Suppl. Tables II, IV, and V) (*P* < 0.001 when compared to the control) whereas the blood glucose levels were within the borderline range (Suppl. Table III). With exercise, the body weight, total cholesterol, total circulating insulin, and blood glucose levels were reduced to near to the control (*P* < 0.001 HFD Ex when compared to HFD, *P* < 0.01 HCD Ex when compared to HCD). Thus, the rat model with prediabetic high circulating insulin was standardized. Exercise training showed a significant role in controlling insulin levels.

### 3.2. Acetyl Cholinesterase Activity in Muscles

AChE is the primary enzyme responsible for the hydrolytic metabolism of the neurotransmitter acetylcholine (ACh) into choline and acetate. AChE found in the neuromuscular junction of the skeletal muscle is synthesized by the muscle rather than the nerve cell. AChE activity was measured at different substrate concentrations. The results of rate measurements as a function of acetylcholine concentration are illustrated, which show data obtained at 30 min incubation at 37°C. Activity was maximum at 1.5 mM concentration. AChE activity was significantly decreased in HFD and HCD rats (*P* < 0.001) when compared to the control whereas the exercise group showed significantly higher activity (*P* < 0.001) when compared to HFD and HCD, respectively (Table 1). With exercise, the skeletal muscle activity was increased thereby enhancing the AChE activity.

### 3.3. Analysis of LDH and Lactate

The optimal sodium pyruvate concentration for the measurement of LDH activity in muscles was estimated to be 1 mM at 25°C. The exercise groups showed significantly higher activity (*P* < 0.01) compared to control rats. The hyperinsulinemic groups also showed an increased LDH activity (*P* < 0.05; Table 2). Our study showed an increase in the total lactate levels in hyperinsulinemic rats compared to control rats. With exercise, the lactate levels were reduced (*P* < 0.01; Table 2). This shows that even though the LDH activity is increased in hyperinsulinemic and exercised rats compared to the control, the lactate formed is cleared by exercise whereas lactate accumulation occurs in hyperinsulinemic cases.

### 3.4. Insulin Signaling in Skeletal Muscle

Insulin resistance indicates an inadequate strength of insulin signaling from the InsR downstream to the final substrates of insulin action involved in multiple metabolic and mitogenic aspects of cellular function. Physical exercise promotes glucose uptake and makes the tissues more sensitive to insulin. To understand the role of the insulin signaling pathway, we evaluated the role of InsR and IRS1 during insulin-resistant and exercise states using anti-InsR and anti-IRS1 serine antibodies in rat muscle tissues.

#### 3.4.1. Insulin Receptor Expression

**Figure 1:**
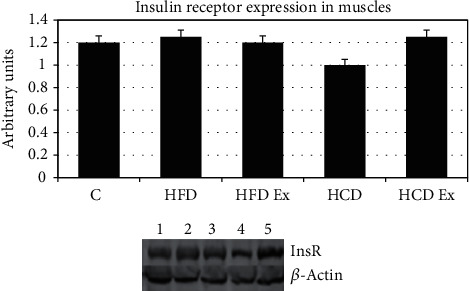
Protein expression of InsR in the muscle. Values are mean ± SEM of 4–6 separate experiments (*n* = 10‐12 animals per group). No significant difference across the group. Lane 1: C (control), lane 2: HFD (high-fat diet), lane 3: HFD Ex (high-fat diet with exercise), lane 4: HCD (high-carbohydrate diet), and lane 5: HCD Ex (high-carbohydrate diet with exercise).

The total InsR expression was studied in the muscle tissue. Our result revealed no significant changes in the InsR expression in muscle tissues of the control, HFD, HCD, and exercise group rats (Figure 1). This shows that the expression of overall number of InsRs is not affected with the high fat and carbohydrate diet or exercise. These results lead us to further analyze the phosphorylation sites on the IRS1.

#### 3.4.2. Tyrosine p y612 Expression

**Figure 2:**
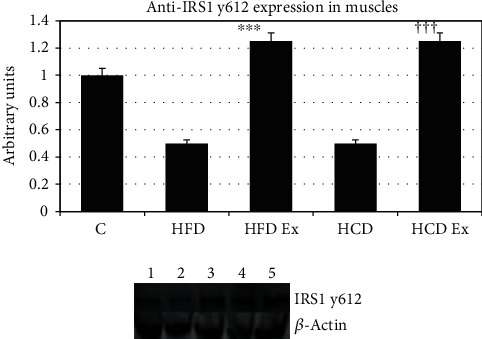
Protein expression of IRS1 tyrosine p y612 in the muscle. Tyrosine p y612 is the PI3 docking site; phosphorylation activates the downstream process in insulin signaling pathways. Values are mean ± SEM of 4–6 separate experiments (*n* = 10‐12 animals per group). ^∗∗∗^*P* < 0.001 when compared to HFD, ^†††^*P* < 0.001 when compared to HCD. Lane 1: C (control), lane 2: HFD (high-fat diet), lane 3: HFD Ex (high-fat diet with exercise), lane 4: HCD (high-carbohydrate diet), and lane 5: HCD Ex (high-carbohydrate diet with exercise).

Tyrosine p y612 is the PI3 docking site. Phosphorylation of this site activates the downstream process in the insulin signaling pathway. In the present study, we found that tyrosine p y612 phosphorylation was significantly decreased in the muscles of HFD and HCD rats and exercise produced significantly increased phosphorylation (^∗∗∗^*P* < 0.001 when compared to HFD, ^†††^*P* < 0.001 when compared to HCD) indicating that the insulin signaling pathways are active in exercise groups (Figure 2).

#### 3.4.3. Serine p636/639 Expression

**Figure 3:**
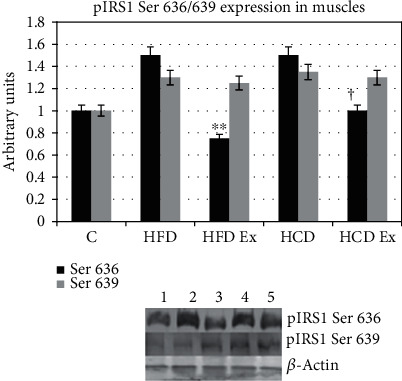
Protein expression of IRS1 serine 636/639 in the muscle. On phosphorylation of serine p-636/639, it inhibits the insulin signal transduction. Values are mean ± SEM of 4–6 separate experiments (*n* = 10‐12 animals per group). ^∗∗^*P* < 0.01 when compared to HFD, ^†^*P* < 0.05 when compared to HCD. Ser 639 showed no significant difference across the group. Lane 1: C (control), lane 2: HFD (high-fat diet), lane 3: HFD Ex (high-fat diet with exercise), lane 4: HCD (high-carbohydrate diet), and lane 5: HCD Ex (high-carbohydrate diet with exercise).

Phosphorylation at serine 636 and 639 is inhibitory to insulin signal transduction. Using a specific anti-phospho-Ser 636 antibody, we found that the IRS1 phosphorylated on serine 636 was significantly higher in the muscle tissue from HFD and HCD than in the control groups. Exercise decreased the phosphorylation thereby activating the insulin signaling pathway (^∗∗^*P* < 0.01 when compared to HFD, ^†^*P* < 0.05 when compared to HCD; Figure 3). Serine p639 phosphorylation did not show any significant changes among the muscles of experimental rats (Figure 3). This indicates that the inhibition of the insulin pathway might be through serine p636 in muscles of rats.

#### 3.4.4. GLUT4 Expression

**Figure 4:**
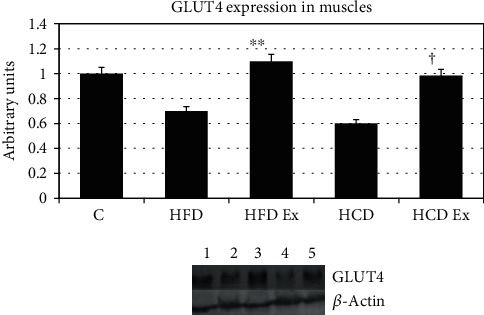
Protein expression of GLUT4 in the muscle. GLUT4 is the main glucose transporter in muscles. Values are mean ± SEM of 4–6 separate experiments (*n* = 10‐12 animals per group). ^∗∗^*P* < 0.01 when compared to HFD, ^†^*P* < 0.05 when compared to HCD. Lane 1: C (control), lane 2: HFD (high-fat diet), lane 3: HFD Ex (high-fat diet with exercise), lane 4: HCD (high-carbohydrate diet), and lane 5: HCD Ex (high-carbohydrate diet with exercise).

GLUT4 is the major transporter in muscles. Expression of GLUT4 was decreased in the muscles of HFD and HCD rats. Exercise increased the expression of GLUT4 (^∗∗^*P* < 0.01 when compared to HFD, ^†^*P* < 0.05 when compared to HCD) indicating an enhanced glucose utilization (Figure 4).

### 3.5. Estimation of Rat Inositol 1,4,5 Triphosphate (IP3) Diacylglycerol Acid, Cyclic AMP, and Cyclic GMP

**Figure 5:**
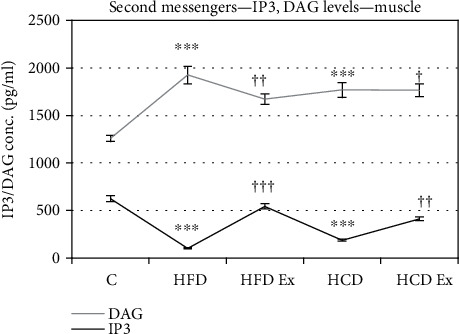
ELISA assay showing IP3 and DAG levels in the muscles. Values are mean ± SEM of 4–6 separate experiments (*n* = 10‐12 animals per group). ^∗∗∗^*P* < 0.001when compared to control and ^†^*P* < 0.05,, ^††^*P* < 0.01, and ^†††^*P* < 0.001when compared to HFD and HCD, respectively. C: control; HFD: high-fat diet; HFD Ex: high-fat diet with exercise; HCD: high-carbohydrate diet; HCD Ex: high-carbohydrate diet with exercise.

**Figure 6:**
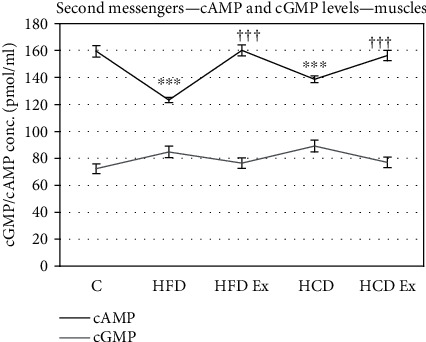
ELISA assay showing cAMP and cGMP levels in the muscles. Values are mean ± SEM of 4–6 separate experiments (*n* = 10‐12 animals per group). ^∗∗∗^*P* < 0.001 when compared to control, ^†††^*P* < 0.001 when compared to HFD and HCD. No significant differences among the group in cGMP levels. C: control; HFD: high-fat diet; HFD Ex: high-fat diet with exercise; HCD: high-carbohydrate diet; HCD Ex: high-carbohydrate diet with exercise.

IP3 and cAMP levels were significantly lowered in hyperinsulinemic groups whereas DAG levels were increased significantly compared to the control (^∗∗∗^*P* < 0.001 when compared to the control). With exercise, the IP3 and cAMP levels were considerably increased whereas DAG levels were lowered (^†^*P* < 0.05, ^††^*P* < 0.01, and ^†††^P < 0.001 when compared to HFD and HCD). But not much difference in the levels of cGMP was observed across the groups (Figures 5 and 6).

## 4. Discussion

With the increasing rate of diabetes worldwide, it becomes necessary to implement strategies to identify high-risk populations and delay or prevent diabetes onset. With altered food habits, sedentary lifestyle, and obesity as key contributors, type 2 diabetes is alarmingly increasing in children and adolescents. Several studies propose that the relationship between diabetes and its complications—CVD, fatty liver, stroke, etc.—begins early and is often associated with hyperinsulinemia [27]. Various clinical studies from our hospital showed that high insulin resistance and high fasting plasma insulin levels can be found at an early age. High insulin resistance and plasma insulin is observed in patients even before hyperglycemia is evident [20]. Participation in regular physical activities is shown to prevent or delay the onset of type 2 diabetes [28]. The present study for the first time tried to understand the changes occurring in the insulin signaling pathway and the metabolic dysregulations occurring during the development of hyperinsulinemia and the effect of exercise on these signaling and metabolic pathways. An intervention done during an early hyperinsulinemic stage can have high beneficial effects than after the development of diabetes.

Indian diet is very high in carbohydrates (70-75%) and fat. Several studies have shown that a high intake of carbohydrate may lead to hyperinsulinemia, high serum TAG, and low HDL-cholesterol levels associated with insulin resistance. Also, HFD have been indicated in the development of hepatic and peripheral tissue insulin resistance in rats [29–31]. Although it is known that high fat or sucrose feeding leads to insulin resistance, nobody has ever investigated whether the type of diet triggers different molecules in the insulin signaling pathway. Here, we established prediabetic hyperinsulinemia in rats by feeding a high fat and carbohydrate diet, resulting in higher body weight, serum insulin level, and total cholesterol levels compared to the control whereas the glucose levels were within normal limits (Suppl. Tables II–V). The hyperinsulinemic stage in this study mimics the hyperinsulinemic state seen in the early stages of diet-induced type 2 diabetes.

Next, our aim was to examine the effect of hyperinsulinemia and exercise on the AChE activity in muscle tissue. Neuromuscular transmission is involved in type 2 diabetes, contributing to different alterations in the physiology of skeletal muscles leading to common clinical complaints of muscle weakness and fatigue. Several tissues show considerable differences in total AChE activity in diabetes reflecting the dysfunction of the cholinergic system during this disease [32]. Our study showed a diminished activity of AChE in HCD and HFD-fed rats which indicate cholinergic system dysfunction. When AChE activity decreases, ACh is not broken and accumulates within synapses which therefore cannot function in a normal way [33]. Next, we aimed to examine the metabolic dysregulations during hyperinsulinemia. Traditionally, an increase in LDH activity has been used as an indicator of defective oxidative capacity related to vigorous exercise and hypoxia [34]. However, in our situation, LDH activity was increased not only because of exercise but also in reaction to the hyperinsulinemic state. But increased lactate levels were found only in rats fed with high fat and carbohydrate diets, whereas in exercised rats, the lactate levels were within normal limits. So, it can be said that during hyperinsulinemia, anaerobic glucose metabolism is activated due to exaggerated glycolysis, producing excessive pyruvate that is further converted to lactate leading to high circulating lactate levels finally causing lactic acidosis and related problems. But the exact connecting link between high insulin and lactate levels is not clearly understood. This high lactate levels along with high insulin can be considered as a triggering factor for the further progression of hyperinsulinemia to type 2 diabetes and related complications [34, 35].

Insulin regulates skeletal muscle metabolism by promoting glucose transport, glycogen synthesis, and protein synthesis through the PI3K/AKT signaling pathway [36]. The role of InsR and IRS1 phosphorylation and the association/activation of PI3K with IRS1 in the muscle of prediabetic rats were studied. Since IRS1 undergoes tyrosine phosphorylation and associates with the PI3K during insulin stimulation, IRS1 may be considered as an important intermediate in insulin signal transmission. It is usually observed that in most patients, the defects lie at the post receptor level of insulin signaling. Though functional deficiencies in IRS1 have been identified in type 2 diabetic patients with normal levels of InsR [37], not much study has been carried out looking at the IRS1 phosphorylation pattern during the early hyperinsulinemic stage. Our results with the InsR expression were similar in all experimental groups indicating that the receptor numbers do not play a significant role for a substantial reduction in insulin action during insulin-resistant states. IRS1 has multiple potential serine, threonine, and tyrosine phosphorylation sites. Studies have shown that increased serine/threonine phosphorylation of IRS1 affects the phosphorylation on tyrosine residues, thereby affecting downstream events of insulin signaling. Ser/Thr phosphorylation serves as a negative feedback mechanism to positive IRS signaling through tyrosine phosphorylation [38–40]. We also provide confirmation for a decreased tyrosine phosphorylation of IRS1 and significant higher phosphorylation of IRS1 on serine 636 and 639 during hyperinsulinemic states. It is thus evidenced that the serine phosphorylations prevent the tyrosine phosphorylation of IRS1 and the subsequent association of IRS1 with PI3K in the skeletal muscle tissue. PI3K is necessary for insulin-stimulated translocation of GLUTs. Tyrosine p y612 is the PI3 docking site which further stimulates translocation of GLUTs [41]. Bouzakri and coworkers found that serine 636 phosphorylations on IRS1 were significantly higher in muscle cells from diabetes patients than in cells from control subjects. MAPKs play a role in the phosphorylation of serine 636/639 of IRS1 resulting in the defective activation of the PI3K by insulin in cells from patients with diabetes [42, 43]. In skeletal muscle cells, the high-affinity GLUT4 is translocated from internal vesicles to the plasma membrane in response to glucose uptake signals [44]. Thus, we found that feeding a high fat or carbohydrate diet resulted in defective PI3K activation causing a selective downregulation of GLUT4 in the muscle. Thus, HFD and HCD can result in severe metabolic and insulin signaling pathway dysregulation.

Defective cellular calcium handling further augments the problems during insulin resistance. Activation of PLC induces hydrolysis of phosphatidylinositol-bisphosphate (4,5) to inositol 1,4,5-trisphosphate (IP3) and diacylglycerol. IP3 acts as a second messenger that mobilizes calcium from intracellular stores via activation of specific IP3 receptors, whereas a major function of diacylglycerol is to activate protein kinase C. cAMP activates PKA, enabling it to phosphorylate IP3R and induce its activity [45–47]. Our study showed a decrease in IP3 and cAMP levels with increased DAG levels during hyperinsulinemia which is an indication of defective calcium homeostasis. Disruptions in calcium homeostasis might be linked to diminished insulin responsiveness. Accumulation of diacylglycerol can result in phosphorylation of serine and/or threonine residues of IRS, leading to impaired PI3K and Akt activity and decreased glucose uptake [48].

Chronic exercise for 2 weeks in rats helped to reduce the body weight, high cholesterol, and insulin levels (Suppl. Tables II–V). Also, we observed a significant increase in AChE activities after exercise indicating that exercise could elicit the AChE by increasing the muscle activity. There are several studies elucidating the influence of exercise on the cholinergic system [49]. All the signaling defects seen during prediabetic insulin-resistant states were reversed with regular vigorous exercise shown by increased expression of GLUT4 muscles. Serine 636 expressions were reduced whereas the tyrosine p y612 expression was increased with exercise. The reduction of serine phosphorylation in rats submitted to exercise was accompanied by increased insulin signaling and correlates with increases in tyrosyl phosphorylation of InsR and IRS1 in muscles. Similarly, the lactate accumulation observed in hyperinsulinemic states was reduced with exercise. The high lactate production was balanced by disposal in exercise-trained rats by greater clearance rates due to increased oxidation and gluconeogenesis of lactate. Transiently elevated lactate obtained during physical exercises improves insulin resistance to peripheral tissues whereas chronical hyperlactatemia might indicate the early stages of prediabetes and contributes to the onset of diabetes. Though there are enormous data showing physical activity as an effective approach for improving insulin resistance by increasing the metabolism of glucose in muscles, there are little studies looking into the effect of exercise on the phosphorylation and metabolic changes due to hyperinsulinemia. Improvement in insulin sensitivity in muscles due to exercises may be indicated by upregulation of the GLUT4 expression and chronic activation of facilitation of insulin signal transduction at the level of PI3K and AS160, as well as increases in the expression of several proteins involved in glucose transport [50–52]. Thus, understanding the precise underlying biological mechanisms mediated during exercise can aid in the development of proper therapeutic interventions.

Combining the clinical observations with our present molecular work, we would like to emphasize the fact that hyperinsulinemia rather than hyperglycemia is responsible for the development of complications like CVD, fatty liver, stroke, etc. Therefore, an intervention during an early stage can improve the quality of life and minimize the diabetic complications. Moreover, long-term insulin treatment lowers glucose and results in increased risk of hypoglycemia. Studies by Joseph et al. showed that insulin-induced hypoglycemia causes more damage to the brain at the molecular level than the streptozotocin-induced hyperglycemic condition [53]. The enhanced neurodegeneration in hypoglycemia is suggested to have more impairment of the motor learning and memory ability which has clinical significance in diabetes treatment. Hence, long-term management of diabetes type 2 with regular insulin therapy may not be beneficial. Our previous work in heart tissue shows that long-term exposure to hyperinsulinemia might cause vascular damages leading to CVDs. Also lifestyle management could help in prevention/delay of further development of hyperinsulinemia to T2DM and further complications [21, 22]. So the therapeutic interventions should focus on lowering the high insulin levels than the high glucose levels. Furthermore, high lactate levels can be used to calculate the risk of diabetes incidence in the future. Our study confirms that adhering to chronic/regular exercise training from the prediabetic stage lowered the high insulin levels and improved insulin sensitivity thereby delaying the onset of vascular complications. Since our clinical and molecular biology studies revealed that high insulin resistance and high fasting plasma insulin even in the absence of hyperglycemia eventually leads to development of CVD, fatty liver, stroke, and diabetes, the early detection and intervention is vitally important, and hence, fasting insulin level estimation should be included in the health checkups along with glucose estimation. Also, regular physical exercise and avoidance of obesity will reduce both glucose and insulin levels and may be beneficial in both normal people and those with diabetes.

## 5. Conclusion

Our molecular data showed that diet-induced prediabetic hyperinsulinemia resulted in impaired insulin signaling and multiple postreceptor intracellulardefects including impaired glucose transport and metabolism. The most effective approach to improve insulin resistance and to reduce cardiovascular risk is appropriate changes in lifestyle. Hence, our study reinforces the importance of regular exercise, as the therapeutic management of insulin resistance by slowing or reversing the progression to diabetes and reducing the morbidity and mortality due to complications associated with diabetes.

## Figures and Tables

**Table 1:** Effect of diet-induced hyperinsulinemia and exercise on acetyl cholinesterase activity.

	*V*_MAX_ (*μ*mol min^−1^/*μ*g protein)	*K*_*M*_ (mM)
Control	6.50 ± 0.12	0.75 ± 0.10
HFD	3.81 ± 0.11^†††^	0.60 ± 0.08
HFD Ex	9.92 ± 0.08^∗∗∗^	1.39 ± 0.08^∗∗^
HCD	3.95 ± 0.15^†††^	0.88 ± 0.04
HCD Ex	8.95 ± 0.13^∗∗∗^	1.33 ± 0.09^∗∗^

Effect of diet-induced hyperinsulinemia and exercise on acetyl cholinesterase activity (km, mM; *V*_max_, *μ*mol min^−1^/*μ*g protein) in muscles at 1.5 mM substrate concentration after 30 min incubation of control, high-fat diet (HFD), high-fat diet exercise (HFD Ex), high-carbohydrate diet (HCD), and high-carbohydrate diet exercise (HCD Ex). For determination of substrate kinetics of AChE, acetylthiocholine iodide was used as the substrate over a concentration range of 0.5– 5 Mm. Values are mean ± SEM of 4–6 separate experiments (*n* = 10‐12 animals per group). ^†††^*P* < 0.001when compared to the control,^∗∗^*P* < 0.01and^∗∗∗^*P* < 0.001when compared to HFD and HCD, respectively.

**Table 2:** Effect of diet-induced hyperinsulinemia and exercise on lactate dehydrogenase activity and total lactate levels.

	LDH activity *V*_MAX_ (*μ*mol min^−1^/*μ*g protein)	Total lactate levels (mmol/l)
Control	0.51 ± 0.07	1.7 ± 0.3
HFD	1.34 ± 0.08^†^	7.6 ± 1.0^††^
HFD Ex	2.1 ± 0.11^††^	2.2 ± 0.5^∗∗^
HCD	1.2 ± 0.12^†^	5.5 ± 0.4^††^
HCD Ex	1.98 ± 0.17^††^	1.8 ± 0.3^∗∗^

Kinetic activity of LDH enzyme and lactate concentrations in rat muscle homogenate. The rate of oxidation of NADH which is directly proportional to LDH activity was read at the end of 10 minutes among the groups control, high-fat diet (HFD), high-fat diet exercise (HFD Ex), high-carbohydrate diet (HCD), and high-carbohydrate diet exercise (HCD Ex) at 340 nm. Lactate levels determined according to lactate assay kit protocol. Values are mean ± SEM of 4–6 separate experiments (*n* = 10‐12 animals per group). ^†^*P* < 0.05 and ^††^*P* < 0.01 when compared to control, ^∗∗^*P* < 0.01 when compared to HFD and HCD.

## Data Availability

Data is available on request.

## Supplementary Materials

Supplementary Materials Standardization of hyperinsulinemic rat model is included as a separate table in the supplementary data. The control group was fed a standard diet, and the other groups (HFD and HCD) received a high fat or carbohydrate diet. After 4 weeks, the exercise groups HFD Ex and HCD Ex rats, were randomly chosen from the HFD and HCD groups, respectively, and trained on a small animal treadmill running at 20 m/min with 5% inclination for 30 min, 5 days/wk. Diet was maintained for 6 weeks. All rats were familiarized to the treadmill by walking 10 m/min for 10 min on the first day, then slowly increasing the duration to 20 min on the second day and then 30 min on the third day. After becoming familiarized with treadmill running, the rats were trained at 20 m/min for 30 minutes. Blood glucose, total cholesterol, total circulating insulin, and body weight were monitored and recorded periodically. Detailed study timeline and exercise protocol included as Suppl. Figure I and Suppl. Table I. Body weight, total cholesterol, total circulating insulin, and glucose levels were included as Suppl. Tables II, III, IV, and V.
