# Supplementary material for: Hyperinsulinemia Induced Altered Insulin Signaling Pathway in Muscle of High Fat- and Carbohydrate-Fed Rats: Effect of Exercise
Source: J Diabetes Res. 2021 Feb 23;2021:5123241. doi: 10.1155/2021/5123241 (PMC7929694; doi:10.1155/2021/5123241)
Supplement: Supplementary Materials — Standardization of hyperinsulinemic rat model is included as a separate table in the supplementary data. The control group was fed a standard diet, and the other groups (HFD and HCD) received a high fat or carbohydrate diet. After 4 weeks, the exercise groups HFD Ex and HCD Ex rats, were randomly chosen from the HFD and HCD groups, respectively, and trained on a small animal treadmill running at 20 m/min with 5% inclination for 30 min, 5 days/wk. Diet was maintained for 6 weeks. All rats were familiarized to the treadmill by walking 10 m/min for 10 min on the first day, then slowly increasing the duration to 20 min on the second day and then 30 min on the third day. After becoming familiarized with treadmill running, the rats were trained at 20 m/min for 30 minutes. Blood glucose, total cholesterol, total circulating insulin, and body weight were monitored and recorded periodically. Detailed study timeline and exercise protocol included as Suppl. Figure I and Suppl. Table I. Body weight, total cholesterol, total circulating insulin, and glucose levels were included as Suppl. Tables II, III, IV, and V. [file 5123241.f1.docx]

# Research Article

**Hyperinsulinemia induced altered insulin signaling pathway in muscle of high fat and carbohydrate fed rats: Effect of exercise**

Anu Joseph, PhD.*, Parvathy S, Koyikkal Karthikeya Varma,

MIMS Research Foundation, Mankavu P.O., Calicut, Kerala 673007, India

**Abbreviated Title: Effect of Exercise on Hyperinsulinemia**

### **SUPPLEMENTARY FIGURES**

**FEEDING STARTS CONTROL/HIGH FAT /HIGH CARB (6 WEEKS)**

**SACRIFICE**

**EXERCISE STARTS HCD EX / HFD EX (2 WEEKS)**

| ACCLIMATIZATION (2 WEEKS) | WEEK 1 | WEEK 2 | WEEK 3 | WEEK 4 | WEEK 5 | WEEK 6 |
| --- | --- | --- | --- | --- | --- | --- |

**DAY 0 OF EVERY WEEK: Body wt estimation, Blood collection**

SUPPLEMENTARY FIGURE 1: **Study Design and Timeline.** After 2 weeks of acclimatization rats are fed with respective diets for 6 weeks. Control feed for control group, High carbohydrate diet for HCD group and High Fat Diet for HFD group. After 4 weeks HFD Ex and HCD Ex group randomly chosen from HFD and HCD respectively and subjected to exercise for 2 weeks. Rats are sacrificed after an overnight fast and 48 h after the last training session. HCD: High carbohydrate Diet, HFD: High Fat Diet, HCD Ex: High Carbohydrate Diet with Exercise, HFD Ex: High Fat Diet with Exercise

### **SUPPLEMENTARY TABLES**

### Supplementary Table I. Exercise protocol on Threadmill

| WEEK 5 | WEEK 6 |
| --- | --- |
| DAY 1 : 10m/ min - 10 minutes | DAY 1: 20m/min - 30 minutes |
| DAY 2: 10m/min - 20 minutes | DAY 2 :20m/min - 30 minutes |
| DAY 3 : 10m/ min - 30 minutes | DAY 3: 20m/min - 30 minutes |
| DAY 4 : 20 m/min - 30 minutes | DAY 4: 20m/min - 30 minutes |
| DAY 5: 20 m/min - 30 minutes | DAY 5: 20m/min - 30 minutes |
| DAY 6 : 20m/min - 30 minutes | DAY 6: Rest |
| DAY 7: 20m/min - 30 minutes | DAY 7: Rest, Fasted |

Total body weight estimated and blood collected on Day 1 of week 5 and 6. Rats were familiarized to the treadmill by walking 10 m/min for 10 min on first day, then slowly increasing the duration to 20 min on second day and for 30 min on third day and then at 20m/min for 30 min. After 2 weeks, rats were sacrificed after an overnight fast and 48 h after the last training session.

### Supplementary Table II: Total body weight

| **BODY WEIGHT (g)** | | | | | | |
| --- | --- | --- | --- | --- | --- | --- |
| **Animal Status** | **Week 1** | **Week 2** | **Week 3** | **Week 4** | **Week 5** | **Week 6** |
| **Control** | 208±09 | 211±08 | 220±11 | 223±12 | 234±08 | 239±09 |
| **HFD** | 205±11 | 235±10 | 250±05 | 267±12^††^ | 275±12^†††^ | 281±12^†††^ |
| **HFD Ex** | 197±11 | 227±09 | 246±12 | 258±11^††^ | 255±05^†^ | 243±14^###^ |
| **HCD** | 204±08 | 229±12 | 240±11 | 245±08^††^ | 264±13^†††^ | 278±11^†††^ |
| **HCD Ex** | 200±07 | 227±12 | 242±14 | 253±08^††^ | 247±11 | 241±13*** |

Total body weight of Control, High Fat diet (HFD), High fat diet exercise (HFD Ex), High Carbohydrate diet (HCD), High Carbohydrate diet Exercise (HCD Ex). Values are mean ± SEM of 4–6 separate experiments (n=10-12 animals per group). ^††^ (P < 0.01), ^†††^ (P < 0.001) when compared to control. ^###^ (P < 0.001) when compared to HFD. *** (P < 0.001) when compared to HCD.

Supplementary Table III:Blood Glucose Levels

| **BLOOD GLUCOSE LEVELS (mg/dL)** | | | | | | |
| --- | --- | --- | --- | --- | --- | --- |
| **Animal Status** | **Week 1** | **Week 2** | **Week 3** | **Week 4** | **Week 5** | **Week 6** |
| **Control** | 110±5.8 | 108±3.2 | 98±3.0 | 105±1.5 | 115±3.4 | 113±2.5 |
| **HFD** | 99±3.2 | 112±2.6 | 118±2.0^††^ | 120±4.6^††^ | 133±3.8^††^ | 138±2.0^††^ |
| **HFD Ex** | 97±2.6 | 111±3.6 | 120±2.8^††^ | 129±3.2^††^ | 123±1.8^††#^ | 119±4.3^††#^ |
| **HCD** | 95±4.0 | 113±1.3 | 120±4.6^††^ | 124±3.0^††^ | 128±2.3^††^ | 130±3.3^††^ |
| **HCD Ex** | 98±3.5 | 110±3.3 | 123±3.2^††^ | 128±3.5^††^ | 125±1.5^††^ | 120±3.6^††^ |

Blood Glucose Levels of Control, High Fat diet (HFD), High fat diet exercise (HFD Ex), High Carbohydrate diet (HCD), High Carbohydrate diet Exercise (HCD Ex). Values are mean ± SEM of 4–6 separate experiments(n=10-12 animals per group). .^††^ (P < 0.01when compared to control ^#^ (P < 0.005) when compared to HFD.

### Supplementary Table IV :. Total Cholesterol Levels.

| **TOTAL CHOLESTEROL LEVELS (mg/dL)** | | | | | | |
| --- | --- | --- | --- | --- | --- | --- |
| **Animal Status** | **Week 1** | **Week 2** | **Week 3** | **Week 4** | **Week 5** | **Week 6** |
| **CONTROL** | 109±2.4 | 115±4.5 | 114±3.9 | 120±1.0 | 122±3.1 | 134±3.7 |
| **HFD** | 101±2.3 | 120±3.6 | 144±4.3^†††^ | 176±3.2^†††^ | 210±2.2^†††^ | 228±3.8^†††^ |
| **HFD Ex** | 106±3.2 | 128±4.2 | 159±5.8^†††^ | 186±3.2^†††^ | 177±4.1^††† ##^ | 159±4.1^††###^ |
| **HCD** | 104±3.1 | 115±2.1 | 128±1.3 | 146±1.2^†††^ | 169±3.8^†††^ | 189±2.1^†††^ |
| **HCD Ex** | 103±3.5 | 114±3.6 | 153±3.4^†††^ | 179±1.5^†††^ | 141±2.3^†††^** | 135±3.4** |

Total Cholesterol Levels of Control, High Fat diet (HFD), High fat diet exercise (HFD Ex), High Carbohydrate diet (HCD), High Carbohydrate diet Exercise (HCD Ex). Values are mean ± SEM of 4–6 separate experiments(n=10-12 animals per group). ^††^ (P < 0.01), ^†††^ (P < 0.001) when compared to control. ^##^ (P < 0.01) ^###^ (P < 0.001) when compared to HFD. ** (P < 0.01) when compared to HCD.

### Supplementary Table V. Total Insulin Levels

| **TOTAL INSULIN LEVELS (µU/ml)** | | | | | | |
| --- | --- | --- | --- | --- | --- | --- |
| **Animal Status** | **Week 1** | **Week 2** | **Week 3** | **Week 4** | **Week 5** | **Week 6** |
| **CONTROL** | 56±3.4 | 58±3.3 | 52±2.2 | 55±4.5 | 54±2.6 | 59±3.2 |
| **HFD** | 54±2.5 | 57±3.6 | 62±1.9^†^ | 68±3.8^†^ | 82±3.0^†††^ | 88±2.2^†††^ |
| **HFD Ex** | 52±1.5 | 59±2.8 | 65±2.9^†^ | 70±1.3^††^ | 66±1.9^†#^ | 59±3.2^###^ |
| **HCD** | 55±1.8 | 59±1.2 | 65±2.4^†^ | 72±2.6^††^ | 80±3.3^†††^ | 89±1.9^†††^ |
| **HCD Ex** | 48±2.8 | 55±2.6 | 63±1.4^†^ | 71±3.5^††^ | 70±2.3^††^* | 63±1.4** |

Total Insulin Levels of Control, High Fat diet (HFD), High fat diet exercise (HFD Ex), High Carbohydrate diet (HCD), High Carbohydrate diet Exercise (HCD Ex). Values are mean ± SEM of 4–6 separate experiments (n=10=12 animals per group). ^†^ (P < 0.05), ^††^ (P < 0.01), ^†††^ (P < 0.001) when compared to control. ^#^ (P < 0.05), ^###^ (P < 0.001) when compared to HFD. *(P < 0.05), ** (P < 0.01) when compared to HCD.
